# Practical guidance on use of TEARS-Q to diagnose post-stroke emotionalism

**DOI:** 10.1177/02692155211024801

**Published:** 2021-06-17

**Authors:** Niall M Broomfield, Robert West, Mark Barber, David C Gillespie, Allan House, Matthew Walters

**Affiliations:** 1 University of East Anglia, Norwich, Norfolk, UK; 2 Leeds Institute of Health Sciences, University of Leeds, Leeds, West Yorkshire, UK; 3 Glasgow Caledonian University, Glasgow, UK; 4 Western General Hospital, Edinburgh, UK; 5 University of Glasgow College of Medical Veterinary and Life Sciences, Glasgow, UK

**Keywords:** Stroke, neuropsychology, emotionalism

## Abstract

**Objective:**

To evaluate, using a classification tree methodology, the ability of the Testing Emotionalism After Recent Stroke – Questionnaire (TEARS-Q) to determine the need for further assessment of post-stroke emotionalism and to identify those whose emotionalism is sufficiently clear that they need assessment for potential intervention.

**Setting:**

Acute stroke units of nine Scottish hospitals in the context of a longitudinal cohort study of post-stroke emotionalism.

**Subjects:**

A total of 228 stroke survivors recruited between October 1st 2015 and September 30th 2018, within two weeks of stroke.

**Measures:**

The measure was the self-report questionnaire TEARS-Q, constructed based on recognised diagnostic features of post-stroke tearful emotionalism. The reference standard was presence/absence of emotionalism on a diagnostic, semi-structured post-stroke emotionalism interview, administered at the same assessment point.

**Results:**

Nine of 159 subjects scoring 0 or 1 on TEARS-Q were diagnosed with post-stroke emotionalism on the reference standard, compared to 11 of 21 subjects scoring 2 to 5 on TEARS-Q and 42 of 48 participants scoring 6 and above. Adding age, sex, deprivation, stroke type, stroke severity, mood, cognition, daily functioning and education did not improve the prediction accuracy sufficiently to change the classification tree.

**Conclusion:**

TEARS-Q reliably identifies those who need no further post-stroke emotionalism assessment, those who need further assessment to clarify diagnosis, and those who almost certainly have post-stroke emotionalism and may benefit from intervention.

## Introduction

We developed and evaluated a self-reported questionnaire TEARS-Q (Testing Emotionalism After Recent Stroke-Questionnaire).^1^ Post-stroke emotionalism is characterised by lessening of control of emotional expression, in particular unheralded crying episodes.^2^

We constructed TEARS-Q based on post-stroke emotionalism crying diagnostic criteria: (i) increased tearfulness, (ii) crying comes on suddenly, with no warning, (iii) crying not under usual social control, (iv) crying episodes occur at least once weekly.^3^ The reference standard was a diagnostic, semi-structured post-stroke emotionalism interview (Testing Emotionalism After Recent Stroke-Interview, TEARS-IV), administered at the same assessment point to *N* = 228 participants recruited within two weeks of stroke to a longitudinal post-stroke emotionalism cohort study (TEARS: NRS Stroke Research Network ID 18980; https://www.stroke.org.uk/research/understanding-difficulty-controlling-emotions-after-stroke).

TEARS-Q has good internal consistency (Cronbach’s alpha 0.87); acceptable discriminant validity (mean score difference of −7.18 between those diagnosed with post-stroke emotionalism from those without); coherent scale dimensionality (one factor accounts for 57% of item response variance), and diagnostic accuracy using a cut-point of 1.5.^1^

Having established psychometric performance based on a single cut point, we sought more detailed evidence to inform the use of TEARS-Q in clinical practice. This was determined by means of a simple classification tree.

## Methods

The TEARS study was approved by Scotland A Research Ethics Committee (IRAS Reference 157483).

This report involves an analysis of the data used in the original study,^1^ and only brief background details will be given here. Participants were male or non-pregnant female, ⩾18 years of age, recruited up to two weeks after a clinical diagnosis of ischaemic or haemorrhagic stroke from acute stroke units of nine Scottish hospitals 2015–2018.^1^ Individuals with aphasia were excluded.

The reference standard was a standardised diagnostic interview based upon recognised features of emotionalism, comprising sections on post-stroke crying and laughing screening questions, frequency and impact and a diagnostic summary. Stroke research nurses, trained by NB and AH, delivered the diagnostic interview.

The index test was TEARS-Q, constructed based on post-stroke emotionalism crying diagnostic criteria, with all eight items valenced identically and a five-point Likert scale to detect positive evidence of symptoms: ‘Strongly Agree, Agree, Unsure, Disagree, Strongly Disagree’, scored 2,1,0,0,0 with maximum total score of 16. The instructional set orients respondents to focus on changes to crying since their stroke, in the past two weeks. Help to complete was offered to a minority of the sample, primarily by reading out questions verbatim.

A classification tree methodology was used to predict post-stroke emotionalism determined by diagnostic interview given the TEARS-Q total scores and other participant characteristics (age, sex, deprivation on Scottish Index of Multiple Deprivation rank,^4^ education level, stroke severity on National Institutes of Health Stroke Scale,^5^ stroke type on Oxford Stroke Classification,^6^ anxiety and depression on Hospital Anxiety and Depression Scale,^7^ cognition on Abbreviated Mental Test^8^ and daily functioning on Barthel Activities of Daily Living Index).^9^ A classification tree provides a set of branching/splitting rules, starting with the entire patient cohort and using each of the variables. The analysis yields two groups, one with a higher prevalence of diagnosed emotionalism than the other, with the split decided by the most statistically significant test from all variables and all the possible cut points (for continuous variables). The two groups are then split further and splitting stops when statistical significance is not reached. The advantage of this approach is that the classification tree selects optimum cut points (plural) for TEARS-Q and the results provide accuracy for a given range of scores, the modelling software R for which has in-built significance tests to determine cut points.^10,11^

## Results

Scores from *N* = 233 participants were available but due to missing data, a final sample of *N* = 228 is reported. Mean age for the sample was 65.0 years (SD 14.6) and 97 of the participants were female. Of the 233 patients, 205 participants had sustained ischemic stroke and 61 were classified as mild stroke on National Institutes of Health Stroke Scale^4^ using a cut-off of 5.

**Figure 1.**
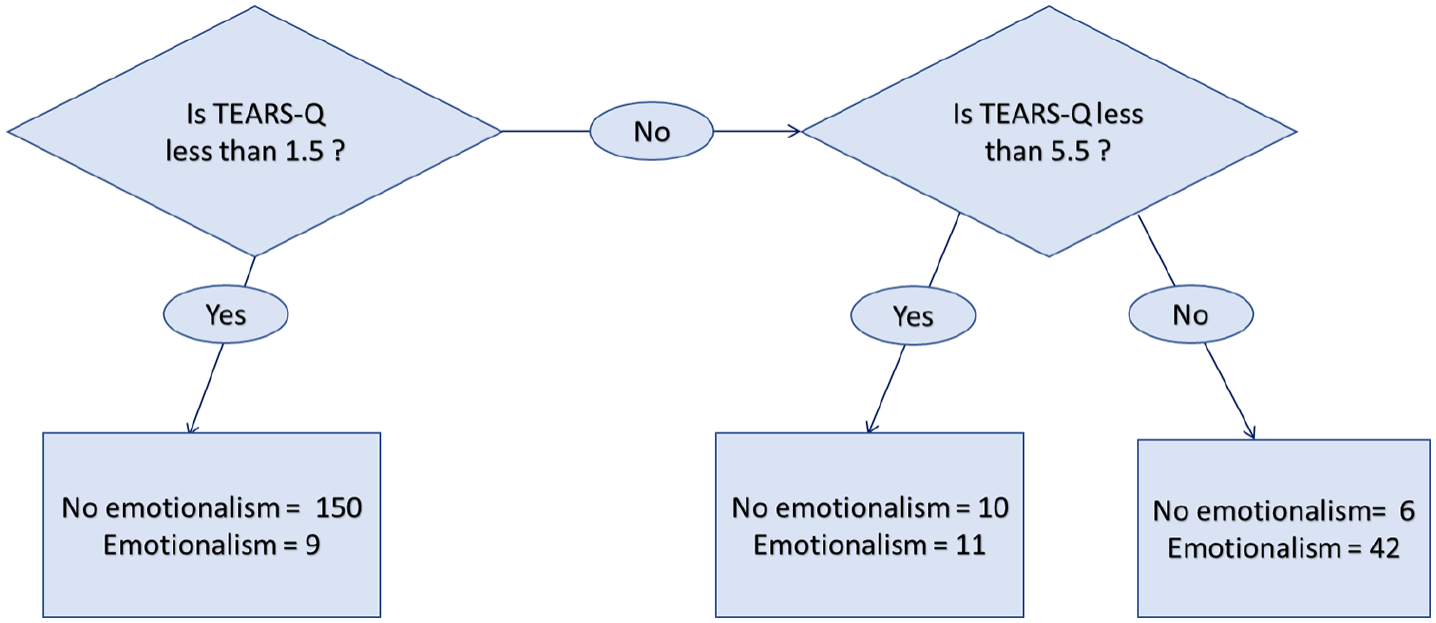
TEARS-Q fitted classification tree.

The fitted classification tree is shown in Figure 1 with cut point determined with 5% significance. Amongst participants with TEARS-Q scores of 0 or 1, 9 of 159 (6%) were diagnosed with post-stroke emotionalism on the reference standard. Amongst participants with TEARS-Q scores of 2, 3, 4 and 5, 11 of 21 (52%) were diagnosed with post-stroke emotionalism. Amongst participants with TEARS-Q scores of 6 and upwards, 42 of 48 (88%) were diagnosed post-stroke emotionalism.

Note that the cut point of 1.5 is identical to that which optimised diagnostic accuracy in our original validation work.^3^ Only the TEARS-Q total score was employed in the fitted classification tree: that is once TEARS-Q is known, adding demographic data of age, sex, deprivation and education did not improve the prediction accuracy sufficiently to change the classification tree.

## Discussion

The classification tree established three ranges of scores on TEARS-Q. The lowest range [0–1] reflects low likelihood of post-stroke emotionalism, so that patients can be screened out. The highest range [6–16] reflects a very high probability of post-stroke emotionalism and patients can be screened in, for consideration of intervention. The middle range [2–5] reflects uncertainty. For patients with scores in this range it is recommended that a full standardised diagnostic interview is conducted. In this sample, only 21/228 (9%) would have needed a full clinical assessment.

Testing Emotionalism After Recent Stroke – Questionnaire (TEARS-Q) is the first self-report questionnaire developed for tearful emotionalism in stroke. Our previous research demonstrated acceptable psychometrics for TEARS-Q which eliminates the need for reliance in stroke settings on previous emotionalism measures, not developed to assess tearful emotionalism in stroke populations. The data reported here build on this, by supporting the use of TEARS-Q to identify those who need no post-stroke emotionalism assessment or intervention, and those who do, both in research and in clinical practice.

There are several limitations to this study. The final TEARS-Q items were not co-produced by, or sense-checked by, a stroke survivor with emotionalism. The reference standard diagnostic interview was developed in the absence of any published, standardised interview schedule. Clinical diagnosis of post-stroke emotionalism could have been an alternate reference standard, but with the disadvantage it would not have been standardised. Participants with aphasia did not complete the assessments, limiting the generalisability of findings. Also, clinical information was available to the nurses who delivered both TEARS-Q and the reference standard, with minimal time interval in between administrations (same day). Nearly 6% of participants who had emotionalism on the interview were not picked up by TEARS-Q. The actual number of missed cases is not absolute however and will depend on the prevalence (prior probability) of emotionalism. Finally, the participants are the same group of individuals involved in the initial TEARS-Q evaluation study.^1^ The sample is relatively young, secondary educated with a bias toward mild stroke, and primarily resident in the West of Scotland. The actual sensitivity and specificity of this method will depend upon the prevalence of emotionalism in a sample. Our sample was reasonably representative of people seen in Scottish stroke rehabilitation services.

Replication using a broader UK population will, therefore, be needed across the whole stroke severity range, in other countries and cultures and including independent administration of TEARS-Q and the reference standard. High-quality research is still needed to establish whether anti-depressant medication is a safe and effective treatment for post-stroke emotionalism.^12^ TEARS-Q has potential as a validated emotionalism outcome measure in the clinical trial context, and to evaluate the impact of non-pharmacological emotionalism interventions.^13^ Research is also needed to develop observational and aphasia suitable TEARS-Q versions, and a parallel measure that can reliably detect and inform treatment planning for (much rarer) uncontrollable laughter emotionalism presentation.^2^

By establishing acceptable psychometrics for TEARS-Q in our previous work, clinically we have eliminated reliance, in stroke settings, on previous emotionalism measures which were not developed to assess tearful emotionalism in stroke populations. The present data build on this by informing guidance, based on the outcome of a brief self-report emotionalism measure, as to which patients may require no further emotionalism assessment, which patients may require more detailed emotionalism assessment and which patients almost certainly have post-stroke emotionalism and may benefit from intervention. Further work in other settings is required, and whether targeted or universal patient screening is deployed (and we would recommend the latter), TEARS-Q could be incorporated into emotionalism care pathways in order to improve stroke care.

**Clinical message**

Using the TEARS-Q screen questionnaire, two thirds (42/62) of patients with emotionalism were identifiable without full assessment, nine cases (out of 228 patients) were not detected, and 21/228 would have needed clinical assessment to detect the remaining 11 cases. The actual number of cases per category, including missed cases, is not absolute and will depend on the prevalence (prior probability) of emotionalism.
